# Open Questions on the Electromagnetic Field Contribution to the Risk of Neurodegenerative Diseases

**DOI:** 10.3390/ijerph192316150

**Published:** 2022-12-02

**Authors:** Joanna Wyszkowska, Colin Pritchard

**Affiliations:** 1 Department of Animal Physiology and Neurobiology, Faculty of Biological and Veterinary Sciences, Nicolaus Copernicus University in Toruń, Lwowska 1, 87-100 Toruń, Poland; 2 Faculty of Health and Social Sciences, Bournemouth University, Bournemouth Gateway, 106 St. Pauls Rd, Bournemouth BH88AJ, UK

**Keywords:** EMF, neurological mortality, early onset dementias, nervous system, environmental pollutants, occupational exposure, Alzheimer’s disease, oxidative stress

## Abstract

This work presents the current state of knowledge about the possible contributory influence of the electromagnetic field on the occurrence of neurodegenerative diseases such as Alzheimer’s and Parkinson’s disease, amyotrophic lateral sclerosis, and multiple sclerosis. Up-to-date literature indicates both favourable and adverse effects of electromagnetic exposure on human health, making it difficult to come to valid and unambiguous conclusions. The epidemiological data analysis from the World Health Organization statistics shows a substantial rise in neurological mortality compared with rises in total populations in developed countries over a mere 15-year period. The largest of the analysed countries produced odds ratios of >100%. The contribution of electromagnetic exposure to the incidence of neurodegenerative diseases is still undoubtedly open to discussion, and it requires further in-depth research to assess the action mechanism of electromagnetic fields in neurodegenerative diseases. The limitations of research published hitherto and the problem of drawing unequivocal conclusions are also in focus.

## 1. Introduction

The increasing number of man-made sources of electromagnetic field (EMF) raises interest in occupational groups about its impact on human health, especially concerning the high level of exposure. While there are some beneficial and therapeutic applications of EMF, there are more and more publications devoted to the unfavourable effects of EMF exposure on humans, mostly pointing to the deterioration of their well-being, disruptions to the functions of the nervous system, or linking it to the occurrence of cancer [1,2,3,4,5,6,7,8].

Many functions of the human body are controlled by electric potentials and currents: the transmission of electric signals in the neuromuscular system, the blood flow associated with the movement of charged particles, and membrane transport phenomena all depend on electric charges and potentials [9]. Thus, EMF has many potential targets for action. However, to fully understand the characteristics of EMF influence, it is necessary to know the exact underlying mechanisms.

Various research is being conducted to determine the effectiveness and safety of the application of EMF in medical treatment. It is crucial to know how powerful EMF can be without disturbing homeostasis, whether compensatory mechanisms appear and whether the effect of EMF is cumulative [10]. It is also necessary to pay attention to the International Agency for Research on Cancer (IARC) 2B classification for possibly human carcinogenic outcomes from chronic EMF exposure [11]. The classification was based on studies demonstrating an association between two types of brain tumours, glioma, and acoustic neuroma, with exposure to radiofrequency EMF from wireless phones. Although the report found that evidence from the occupational and environmental radiofrequency EMF exposure was inadequate, the conclusion is that there could be some risk. Therefore, it is necessary to keep a close watch for a link between cell phones and cancer risk, especially in the category of heavy users. In the case of power-frequency EMF, only the magnetic component was classified as possibly carcinogenic to humans. In the report, studies conducted on occupational exposure pointed to a possible increased risk of leukaemia, brain tumours, and male breast cancer. However, their interpretation was difficult mainly due to methodological limitations and a lack of appropriate exposure measurements [12].

EMF treatment should be conducted under well-controlled conditions, supported by evidence of biological activity for EMF, which may lead to a positive or negative outcome depending on the exposure parameters. This is to ensure the safety of therapeutic treatments involving EMF, even more so when a direct action of an EMF occurring at one body location may have an indirect effect in another location [13,14,15,16]. According to published results, the most beneficial therapeutic cycle should include 10 to 14 daily treatments. In selected cases, it is advisable to repeat the cycle after four weeks [10].

Reflecting on human biology, we need to bear in mind that the brain is essentially an electro-biochemical organ, so potentially, as EMF passes through the human body, it might be expected that the brain and neurological system will most likely be affected by EMF exposure [3,17]. Special attention is needed for cases of chronic exposure, where conclusions from research into the beneficial effects during short and controlled medical EMF treatments may not apply.

The biological effect of exposure to EMF generally depends primarily on its frequency. This is due to the fact that different frequencies interact with the body in different ways: low-frequency EMF may cause the stimulation of nerves and muscles by induced electric potentials, while a high-frequency field induces thermal effects that may lead to a rise in body temperature. EMF exposure at a frequency exceeding 10 GHz causes EMF energy absorption mainly near the surface of the body [9,18].

There have been articles indicating a relationship between the higher incidence of neurodegenerative diseases (NDD) and increased exposure to EMF [1,2,19,20,21]. Researchers are trying to define a mechanism that could explain this correlation by considering the contribution of oxidative stress, which is closely related to the occurrence of neurological diseases and may be developed under the influence of EMF, among other factors [16,22,23]. However, the key point is that it is not possible to simply split the impact of EMF exposure and multiple other interactive environmental pollutants (e.g., increases in background hormone-disruptive chemicals, air pollution, food additives, and petrochemicals from the motor and air transport), which all overlap and contribute to organism response. Each of these factors may play an important role in people’s lives. Epidemiologic evidence appears to suggest that workers, especially in electrical occupations, may be at an increased risk caused not only by higher levels of EMF exposure but also by exposure to other factors (e.g., metals, chemicals) that may interact with EMF [24].

It should be taken into account that the rise in incidents of neurological diseases is a reflection of the Gompertzian hypothesis, which states that people’s life span is becoming longer, so they are developing more age-related diseases [25]. In other words, it is suggested that the apparent increases in the incidence of NDD are mainly due to demographics, i.e., there are more neurological diseases because there are more elderly people. However, we will present and use the latest epidemiological data, based on World Health Organization (WHO) statistics, updated as of December 2018 [26], which contains evidence that seriously challenges such simplistic reassurances and provides cause for concern. With regard to EMF, there has been a degree of uncertainty as studies have not found any statistically significant negative effects related to EMF exposure [21,27,28], and other research results have even indicated that short-term exposure to low-level EMF can help improve memory in Alzheimer patients [29], and functional and mental status of poststroke patients [30].

This paper is an overview of the results arising from the epidemiological, in vitro, and in vivo studies that investigated whether EMF exposure has an influence on the occurrence of neurodegenerative diseases. A literature search was conducted on the online databases of PubMed and Google Scholar and the official reports of the Scientific Committee on Emerging and Newly Identified Health Risks working for the European Council of the European Union [18] and WHO [31]. The following terms were searched in the online databases individually or in combination: “neurodegenerative disease”, “Alzheimer”, “amyotrophic lateral sclerosis”, “ALS”, “Parkinson’s disease”, “multiple sclerosis”, “MS”, “exposure”, “magnetic field”, “electromagnetic field”. The comprehensiveness of the literature search was verified using reviews and reference lists of other publications.

## 2. Neurodegenerative Diseases (NDD)

Neurodegeneration is the progressive loss of the structure or function of neurons, including their death. There are hundreds of disorders that could be described as neurodegenerative diseases (NDD). They are often associated with deficits in brain function (e.g., memory and cognition, or movement–dependent on the predominant neuronal population impacted). Many of these diseases are rare, but a few are common and include Alzheimer’s disease (AD), Parkinson’s disease (PD), amyotrophic lateral sclerosis (ALS), and multiple sclerosis (MS) (see Table 1 for the summary). They represent one of the gravest health concerns currently affecting developed countries. Specific environmental factors and lifestyle, alone or in combination with genetic susceptibility factors, are considered to play a key role in the pathogenesis of NDD [23,32].

The risk of being affected by an NDD increases dramatically with age. More people living longer means that more individuals are affected by NDD, which is why it is so important to improve our understanding of what causes NDD and to develop new approaches for treatment and prevention [33].

**Table 1:** Overview of common neurodegenerative diseases. Based on data from the public domains [34,35].

Disease	Main Neuropathology	Symptoms
Alzheimer’s disease (AD)	Beta-amyloid deposits and neurofibrillary tangles in the cerebral cortex and subcortical grey matter	- Loss of memory, an inability to learn new things, loss of language function, a deranged perception of space, an inability to perform calculations, indifference, depression, delusions, and other manifestations - These deficits affect patients’ social functioning and make it difficult or impossible for them to carry on with their daily lives - AD is inexorably progressive and fatal within 5 to 10 years
Parkinson’s disease (PD)	Loss of neurons that produce dopamine–a chemical messenger in the brain	- Motor symptoms: rigidity, tremor at rest, slowness of voluntary movement, stooped posture, a shuffling, small-step gait, difficulty with balance - Non-motor symptoms: expressionless face, soft voice, olfactory loss, mood disturbances, dementia, sleep disorders, and autonomic dysfunction, including constipation, cardiac arrhythmias, and hypotension - The most common cause of death is pneumonia
Amyotrophic lateral sclerosis (ALS)	Loss of neurons in the motor cortex (upper motor neurons) and motor neurons in the brain stem and central spinal cord (lower motor neurons)	- Trouble walking or running, writing, speech problems - The majority of patients die, usually from respiratory paralysis, within 2–3 years from the onset of symptoms
Multiple sclerosis (MS)	Inflammatory demyelinating processes in the brain and spinal cord (CNS)	- Numbness or weakness in one or more limbs, electric-shock sensations that occur with certain neck movements, tremor, lack of coordination or unsteady gait, partial or complete loss of vision, often with pain during eye movement, prolonged double vision, blurry vision - Slightly more than two in every five people with MS died from the disease or from complications

## 3. Electromagnetic Field and Neurodegenerative Diseases

Researchers have been looking for environmental factors responsible for the development of NDD. Several reports indicate that exposure to electric and magnetic fields may be associated with an increased risk of NDD. The focus of attention is occupational exposure with a relatively high level of EMF exposure, which may be associated with a significant duration of exposure. Several studies [20,21,36,37,38] have addressed this issue.

### 3.1. Earlier Epidemiological Studies

Based on a thorough analysis of death certificates, it was observed that there is a higher fatality ratio from NDD among people professionally exposed to EMF (e.g., electric power line/cable workers, welders, electricians) than in other professional groups [39]. However, the occurrence of AD and ALS was more strongly associated with EMF exposure than PD [40]. In a similar study [41], a higher mortality rate because of AD in men exposed to the magnetic field was stated; in contrast, in another study, ALS deaths had no connection to magnetic field exposure [39]. However, no clear correlation between the results with the actual level of EMF exposure was revealed. Additionally, researchers’ attention was drawn to the death rate of people inhabiting areas adjacent to high-voltage lines. The authors of this article observed an increased mortality rate due to NDD (in particular AD) in residents living near (<50 m) 220–380 kV power lines [42].

A Swedish study seems to reinforce the evidence for a relationship between occupational EMF exposure and AD, however, it showed elevated risks only for a subgroup of manual workers before the age of 75 [43]. Davanipour et al. [44] studied the possible relationship between EMF exposure and severe cognitive dysfunction. The results indicate that working with EMF exposure (10^−4^–10^−2^ mT) may increase the risk of severe cognitive dysfunction. Smoking and older age (75+) may increase the deleterious effect of EMF exposure [44]. The elevated risks of dementia, motor neuron disease, MS, and epilepsy and lower risks of PD in relation to exposure to EMF (10^−4^–10^−3^ mT) were observed in a large cohort of Danish utility sector employees [45].

On the other hand, a study [46] involving an extensive analysis based on a sample of 30,631 people employed in Danish utility companies did not observe the correlation between PD, AD, or any other diseases of the central nervous system and occupational exposure to EMF (10^−4^–10^−3^ mT). Parlett et al. [47], likewise, indicated no increased rate of mortality from motor neuron disease related to people employed in the electronics sector (~3 × 10^−6^ mT). The conducted cohort study showed only 40 (out of 3,000,000 examined people) deaths from a motor neuron disease during an average of 8.8 years of observation [47]. In the majority of the available data, no association between PD and EMF exposure has been observed [37,39,46].

The reviewed papers indicate a possible relationship between NDD and EMF, though they also emphasise the methodological limitations, and so no consistent results and unambiguous conclusions have been reached. For instance, Ahlbom [19] concluded that there is relatively compelling evidence indicating that electric utility work may be associated with an increased risk of ALS. However, EMF exposure is only one of several possible contributing factors. For AD, the evidence for an association with EMF is relatively flimsy [19]. In 2006, in a meta-analysis including eight studies published between January 2000 and July 2005, the increased risk of AD was confirmed [37]. Zhou et al. [38] conducted a meta-analysis of seventeen epidemiological studies. Although the findings were not consistent, the authors indicated a slight but significant ALS risk increase among those performing jobs related to EMF exposure (50/60 Hz, 3 × 10^−4^–10^−3^ mT) [38]. Gunnarsson and Bodin [48] in their meta-analysis of sixteen studies (1998–2017) showed recently that occupational exposure to EMF (50/60 Hz, 10^−4^–10^−3^ mT) seemed to involve a 10% increase in risk for ALS and AD, though no such indication of risk was found for PD [48,49].

The majority of studies based on death certificate examination indicated no association between EMF exposure and the risk of NDD [41,43]. An elevated risk of AD and ALS was shown in small-scale studies or only for subgroups of 65–75-year-old people or manual workers [38,43,44,47]. Studies of motor neuron disease occurrence based on EMF-level exposure assessment showed less evidence of the EMF effect than those relying on job titles alone [18].

Epidemiological studies on neurological diseases in relation to radiofrequency (100 kHz < f ≤ 300 GHz) EMF exposure show no clear effect, though the evidence is limited. Studies focused on an association between mobile phone use and migraine, vertigo, and the risk of PD and MS [50]. The main problems are conflicting results and methodological limitations [18].

Although the publications regarding the association between EMF and NDD are quite numerous, it is important to note that all those analyses are based solely on death certificates and medical documentation, and therefore demonstrate a certain degree of methodological weaknesses (EMF and other environmental factors lack sufficient characterisation). Numerous external factors, such as the severity of work, physical or mental work, and lifestyle can be determinants with regard to the risk of NDD among different professional groups. Moreover, the studied data from death certificates mostly concern people who lived and worked in the 1970s, 1980s, and 1990s (people subject to occupational exposure to EMF were predominantly physical workers). Such data may lead to conclusions that are inadequate and inapplicable these days.

Epidemiological studies have the advantage of long-term observations, though they still contain limitations, such as the relatively crude EMF exposure assessment and the coexistence of many other factors determining neurophysiological pathologies. On the other hand, the small studies have a low statistical power due to a small number of events. Additionally, as EMF exposure is ubiquitous, it is difficult to find an unexposed reference group, and instead, a quantitative contrast is chosen by comparing low versus high exposure levels. Other limitations of this type of study include the fact that most review articles are based on publications stored in electronic databases, usually PubMed, and only English-language publications are included there. These limitations of epidemiological studies are insufficient to conclude that EMF exposure increases the risk of NDD [18].

### 3.2. Laboratory Experiments

Several studies have shown that EMF exposure modifies physiological and biochemical processes leading to immune cell activation [15], resulting in increased reactive oxygen species (ROS) formation, enhanced phagocytic activity, and increased cytokine release [23,51]. It was shown that EMF can cause mild oxidative stress (increase in ROS and changes in antioxidant levels) in many tissues of the body [16,52,53]. The increase in plasma concentration of pro-inflammatory cytokines and an elevation in blood parameters, such as white blood cells, lymphocytes, hemoglobin, and hematocrit levels in rats exposed to EMF (50 Hz, 7 mT, 24 h) were also demonstrated [15,54].

Inflammation in the central nervous system often occurs in the case of AD, PD, or in the case of chronic neurological disorders. In the review [53], the authors indicate that exposure to EMF can cause redox reactions (50 Hz, 0.1–1.0 mT, 7 days) and the induction of oxidative stress (50 Hz, 2 mT, 3 h) in the rodent brain. This increases the level of free radicals after exposure to EMF (50 Hz, 7 mT, 30 min/day, 10 days), which, in turn, leads to oxidative damage to the lipids in the brain of mice and rats. In the experimental model of a rat exposed to 50 Hz EMF (0.1 and 0.5 mT, 7 days), there was a strong toxic effect disturbing the antioxidant effect. It was shown that exposure to 50 Hz frequency EMF (0.1–1.0 mT, 10 days) affects the antioxidant capacity of enzymes in the brain of both young and old rats. However, in older rats, a large decrease in all major anti-oxidative enzymes was observed, thus indicating an age-dependent greater susceptibility to the induction of oxidative stress as a result of exposure to the EMF [53,55]. The age-related differences in the influence of the EMF appear also in the paper of Ivancsits et al. [56], where the authors established an age-related decrease in DNA repair efficiency of EMF (50 Hz, 1 mT, 15 h)-induced DNA strand breaks [56].

Analysis of the body of experimental evidence reveals that it is still unclear whether or not exposure to microwaves (EMF of frequency > 300 MHz) affects the nervous system, including neurobehavioural disorders, although some of the studies suggest a non-thermal level effect on learning, memory or behaviour. Some studies on NDD have shown evidence of a potential correlation between EMF and the mechanism of neurodegeneration. However, the correlation is not clearly defined and studies cannot explain the precise mechanisms. Further studies of these effects are needed [57,58]. The inconsistencies in neuronal parameters in response to microwaves reveal an indeterminacy in identifying the molecular impacts of EMF, and in discriminating thermal from non-thermal effects [59]. The lack of conclusive evidence stems from the ambiguity regarding exposure, proper protocols, control groups, and dosimetry in many studies. Additional experiments are required to assess whether longer-term exposure could be associated with symptoms [18].

Co-exposures of several factors may have a significant influence on the development of NDD. Deng et al. [60] investigated whether memory impairments produced in mice by chronic aluminium (Al) treatment (200 mg/kg) could be modulated by magnetic field exposure (50 Hz, 2 mT for 4 h/day, 6 days/week). It was found that both aluminium and EMF could have an impact on learning memory and pro-oxidative function in mice by neuronal cell loss and overexpression of phosphorylated tau protein in the hippocampus and cerebral cortex. However, there was no evidence of any association between EMF exposure and aluminium loading [60]. Zhang et al. [61] also used chronic Al treatment as a contributing factor to cognitive function impairment in AD to examine whether or not EMF (50 Hz, 0.1 mT) and Al have synergistic effects on AD pathogenesis. The results showed learning and memory impairment, neuronal cell loss, and high density of amyloid-β (Aβ) in the hippocampus and cerebral cortex in Al treatment rats. EMF exposure had no effect on the pathogenesis of AD induced by Al overload [61]. Contrary results were obtained by Liu et al. [62]. This group investigated EMF exposure (50 Hz, 0.4 mT, 60 d) combined with intraperitoneal D-galactose (50 mg/kg, 42 d) which can cause premature aging and organ decline, and Aβ25–35 hippocampal (5 μL) injection inducing AD-like clinicopathological features. All these factors were implemented to establish a complex rat model and relationship between EMF exposure and AD development. The results showed that EMF partially improved the cognitive and clinicopathologic symptoms of AD rats, which indicates that certain conditions of EMF exposure could delay the development of AD in rats [62]. It should be emphasised that the result offers only the possibility of using EMF exposure with specific parameters in medical treatment, and does not exclude possible contraindications to such therapy or its side effects. Overall, extremely low-frequency magnetic field and radiofrequency EMF were evaluated as possibly carcinogenic to humans (Group 2B). On the other hand, the power-frequency electric field, as well as static electric and magnetic field, was judged “not classifiable” based on “inadequate” evidence from both humans and animals (Group 3) [11,12].

Maaroufi et al. [58] tested the hypothesis of a possible link between an iron overload in the brain and neurodegenerative disorders. They studied whether combined radiofrequency EMF exposure (900 MHz, 0.05–0.18 W/kg, 1 h/day, 21 d) and iron overload (which is neurotoxic and can contribute to learning deficits, etc.) influenced the outcome of spatial cognitive tasks, neurochemistry, and oxidative stress in rats. The results show that rats exposed to EMF displayed impaired exploratory activity, but not in the navigation and working memory tasks. Some changes in dopamine levels in certain brain regions were noted, but not in all parts of the brain. There were no consistent effects on parameters related to oxidative balance in the brain. The iron overload did not exacerbate the effects of radiofrequency EMF exposure [58].

Exposure to EMF may interact with chemical agents by exhibiting an increase or a decrease in the effects of the latter. Nevertheless, due to the small number of investigations available and the large variety of protocols adopted (different chemical treatments and different EMF exposure conditions), it is not possible to draw valid conclusions [18].

The question of whether or not EMF increases the incidence of morbidity in people with a genetic predisposition is still open. Experimental results are inconsistent, e.g., some indicate that EMF induces brain DNA damage [63], while others show that EMF exposure does not result in a significant effect on inflammation-related genes or protein expression in the immune cells [64].

Animal studies on changes relevant to human NDD in the context of EMF exposure are scarce [2]. Both in vivo and in vitro experiments used various models and EMF exposure conditions, mostly acute or short-term (with exposures ranging from a few minutes to several days).

## 4. Evidence for Accelerating Neurological Mortality (NM) 2000–2015

The problem with exploring an environmental impact on human health is that it ignores “individual” epigenetic variations. Discoveries reveal that a wide array of environmental, dietary, behavioural, and medical experiences can significantly affect the future development and health of an individual [65]. Based upon earlier research, the question was asked regarding whether there has been an increase in neurological mortality (NM), outstripping changes in population between 1989 and 2015, and the answer was strongly in the affirmative [66]. In the analysed period, the proportion of the over 75-year-olds in the general population rose substantially, e.g., more than doubling in Japan and Spain, and increasing by more than 50% in another 12 analysed countries [24,26]. This problem also occurs in countries that were not described in the WHO report e.g., in Poland where the proportion of over 75-year-olds in the general population increased by 43% between 2002 and 2015 [67].

Likewise, substantial rises in NM were noted in 11 of the 21 analysed developed countries (DC). One remarkable change was that the USA initially had the 15th highest neurological mortality rate out of 21 countries (1979–1997), but by 2015 it had risen to be the second highest [24,26,66]. The initial explanation for these changes was demographics, namely the Gompertzian hypothesis that more people were now living longer and developing age-related diseases [25]. However, the new phenomena of rises in early-onset forms of dementia, occurring in the last 10 years, were ignored [68,69]. Indeed, supportive charities have been developed to help with this growing problem [70], all of which points strongly towards environmental factors. This does not exclude underlying genetic factors, indeed with a greater understanding of epigenetics, it is now appreciated that environmental changes can trigger underlying genetic predispositions [24,66]. Bearing in mind how long genetic and environmental changes need to make a visible impact upon patterns of human health, this work led to a realisation about the relatively short time in which neurological death rates have been accelerating amongst DC.

Here we present the results of the analysis of the most recent WHO mortality data. available, updated December 2018 [26], which demonstrates the extent of the acceleration of NM in two categories: ‘Nervous Disease Deaths’, which includes the major conditions such as Motor Neurone Disease, PD, MS, etc., and, ‘AD and Other Dementia Deaths’, in 21 analysed DC, over a mere 15 year period, 2000 to 2015 [26,71]. The data show that in every DC the rates of death from general neurological diseases such as Motor Neurone Disease, PD and MS for 55–74-year-olds had a greater increase, rather than from dementias in the same age range. While NM rates fell between 2000 and 2015 in Belgium −3%, Canada −11% and France −28%, in twelve countries a substantial increase was observed (>20%). There were notable rises in the larger countries, such as Germany by 52%, Japan 60%, Sweden 52%, USA 50% and the UK 51%, for the combined sexes, in just 15 years.

The changes in total NM rates in 21 selected DC divided by gender are shown in Table 2. Numbers of total NM are represented via the WHO Age-Standardised-Death-Rates (ASDR) per million (pm). The age-standardized mortality rate is a weighted average of the age-specific mortality rates per 100,000 persons, where the weights are the proportions of persons in the corresponding age groups of the WHO standard population. Standardization by age is important for most health problems.

The ASDR in all age groups rose by more than 50% in 15 DC in this century, reaching the highest value in Germany 125%, Japan 151% and UK 202%. The smallest increase was observed in France and Canada, 8% and 14%, respectively, with an overall average increase of 59% over the period (Table 2).

However, rates per million perhaps do not reflect the practical situation faced by families and public health services regarding conditions that usually are present for more than a decade before death. In Table 3. we compare the population of people aged 55–74 and the total populations of the eight largest analysed DC, which gives a more realistic indication of the increasing rate of NM.

All the countries, including the outliers Canada and France, showed a considerable increase, even in the group of 55–74-year-olds. In terms of total NM, compared with rises in total populations, France and Canada produced an odds ratio of 62% and 59%, respectively, and the other six countries’ odds ratios were >100% (Table 3).

Let us look into the changes in the populations using data from the UK and the USA as an example. In Britain, the 55–74 population rose 25%, but NM cases went from 4650 to 9019 (up 94%), while total NM in the entire population went from 24,601 to 103,550, more than trebling, while the total population rose by just 9% in this century. In the USA, the older age band population (55–74 year-olds) rose by an impressive 58%, but their NM went from 21,818 to 48,047, up 120%. With regard to total NM, they rose from 174,708 to 436,438, up 150%, while the population rose by just 14% [26]. Surely the only word to describe these changes is acceleration and in just 15 years. Clearly, having a greater number of older people increases the risk of more age-related deaths, but for that to happen at such speed there seem to be major environmental influences. Whether EMF plays a considerable role is still an open question, but less so when it has come to be seen as one factor amongst various other interactive environmental pollutants, and possibly a triggering factor. Whatever the likely multiple interactive causes of these changes may be, numbers like these are a matter of great concern, and the authorities need to respond by determining exactly the reasons underlying these rises, and how to make the environment safer.

## 5. Discussion

This work aims to present current knowledge about the influence of EMF on the incidence of NDD, which is becoming progressively common in today’s world. Currently, in the era of rapid technical progress, people are surrounded by devices emitting an EMF, and the number of NDD occurrences is rising. It is perhaps unsurprising then, given this apparent correlation, that researchers are trying to understand whether there is any causation between the two issues. The thesis that EMF increases the risk of NDD deserves thorough and comprehensive research, research that crosses disciplinary boundaries due to the interaction of many environmental factors upon human health.

Studies investigating the possible effects of EMF exposure on NDD are too diverse with regard to applied EMF, the duration of exposure, and the statistical methods to draw any reasonable and satisfactory conclusion [18]. In the case of PD and MS, there is not enough research to determine whether EMF affects their development. However, some scientists cast a shadow of uncertainty claiming that EMF contributes to the formation of oxidative stress in the body, and therefore leads to the incidence of these diseases. However, many studies are indicating the participation of EMF in the development of AD and ALS. Although the results are not consistent, there is an increased risk of AD observed across populations. Undoubtedly, further intensive research is needed to assess the mechanism of EMF acting on NDD. The effects on ROS, lipid peroxidation, and antioxidant defence are among the proposed mechanisms, though none of them has been finally defined. The difficulties with the identification and experimental validation of the EMF influence mechanism are due to the variability of biological responses and a lack of consistency in the findings.

**Figure 1:**
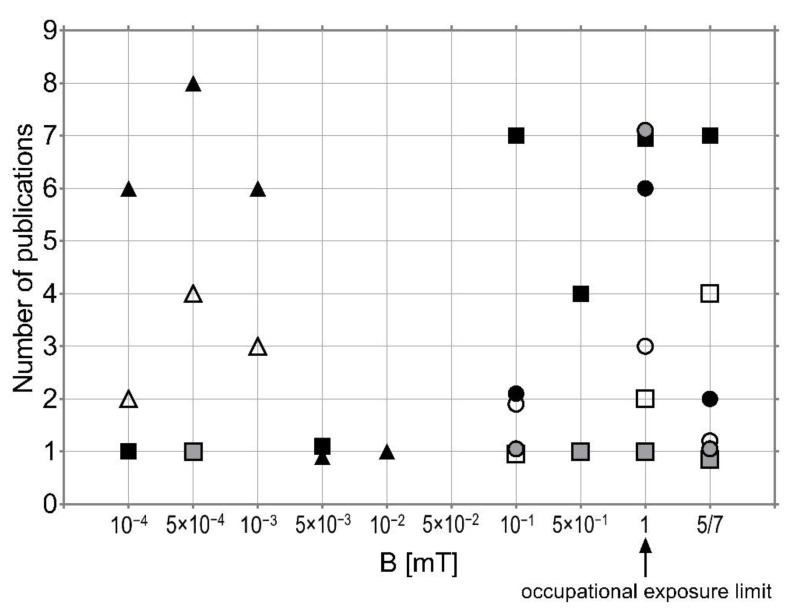
Distribution of EMF effects obtained in epidemiological studies (triangles), animal studies (squares), and in experiments on cells (circles)—reported in publications discussed in this work (for 50/60 Hz): no effects—white, positive—grey, negative—black; reference level for occupational exposure is marked [77].

To summarise, the published results are not unequivocal and are often contradictory (Figure 1), so further research is needed to thoroughly explain the mechanism of action of EMF on the central nervous system, and to explain its potential relationship with NDD. Another important factor that needs to be considered is that the development of a disease does not solely result from environmental factors. As illnesses usually depend on potential genetic predispositions, two individuals exposed to the same noxious pollutant may develop various medical conditions.

In the course of seeking explanations for the impact of EMF on human health, we have concluded that the previous view, whereby most apparent increases in incidences of disease were due to the demographics (having more elderly people in the population), might be flawed. This is clear, not only from the remarkable increase in early-onset-dementias (EOD), but from how, starting in the late 1990s, disproportionate rises in NM have been reported by more and more DC, indicating that the disease process was initiated at an earlier life stage. Of course, having more over 75-year-olds in the overall population involves more age-related diseases, but such NM were often treble the rate of population rises in this age group. So, the question arises–if death is inevitable, why is it due to neurological causes? Perhaps the most thought-provoking finding is the acceleration of NM in a relatively short period of just 15 years. The numbers are alarming, and it would be negligent of us if we failed to emphasise the extent of the problem and the possible contributory causes.

Current scientific data are not sufficient to determine the dependence of the particular effects on the EMF exposure parameters, and thus determine the numerical value of the exposure threshold at which the defence mechanisms of each human body are insufficient (or sufficient) to protect health.

As mentioned, co-exposures of several factors may have a significant influence on the development of NDD. Exposure to EMF may act as an age-dependent risk factor. With age, more and more damaged or misfolded defective molecules are stored in inclusion bodies (“garbage bags”) in and between neurons, thus enhancing the degeneration of cells. The defective molecules also disturb the function of the neurons, leading to cell death [48]. It has been demonstrated that EMF can activate the cellular stress response through increased levels of stress proteins, such as HSP70 [72]. Some authors highlighted that the onset of stress response through EMF exposure should be considered as a defence reaction of the cell to damaging agents [73]. Other authors have suggested the beneficial effects of EMF acting as a mild stressor and inducing protection against various stressors [74,75]. Cellular homeostatic mechanisms may quickly compensate for the physiological disturbances [72]. However, EMF may also decrease the tolerance threshold towards additional oxidative-based challenges. Co-exposure to EMF and other stress factors could trigger the failure of the antioxidant cell response leading to oxidative damage and functional impairment. This, in turn, may significantly increase the risk of the development of NDD [76].

This paper presents the available data on the influence of EMF on the incidence of neurodegenerative diseases and the mechanisms of this impact. The role of EMF as a factor in increased mortality in populations as a result of neurodegenerative diseases is also considered. The work aimed to show how complex this problem is, how difficult it is to compare data with each other, and, therefore, despite the existence of a lot of data, it is difficult to define conclusions. There is no doubt that the impact of EMF on the incidence of neurodegenerative diseases cannot be overlooked and more systematic, standardized research, e.g., using animal models, a well-designed EMF exposure system, and well-defined dosimetry should be conducted.

## 6. Conclusions

Genes, environment, and behaviour significantly determine life expectancy and the types of diseases a person may be plagued with. Environmental and behaviour stressors decide whether a genetic disposition will manifest. To what extent EMF must be counted among those stressors, science cannot say with certainty at this time:

- Studies investigating the possible effects of EMF exposure on NDD are too diverse with regard to applied EMF, the duration of exposure, and statistical methods to draw any reasonable and satisfactory conclusion.
- The difficulties with the identification and experimental validation of the EMF influence mechanism are due to the variability of biological responses and a lack of consistency in the findings.
- There are a number of significant factors besides EMF influencing NDD, such as age, a low level or lack of education, or serious or repeated minor head injuries, and various toxic environmental and occupational agents (including such things as solvents, pesticides, and toxic metals).
- EMF may interact with other multiple environmental pollutants and/or occupational factors.
- EMF may have a beneficial impact as a mild stress factor inducing protection against various stressors or, on the contrary, may disturb the stress response of cells, leading to oxidative damage and functional impairment
- There is no concrete evidence of the positive or negative effects of EMF, however, research should still be carried out in this field, so as not to overlook such a risk factor.

## Figures and Tables

**Table 2:** Male and female combined neurological mortality (NM) in Age-Standardised Death Rates (ASDR), split by sex rates per million (pm) in selected developed countries. Based upon WHO [26] data.

	Country	Total Male NM ASDR		Total Female NM ASDR	
		NM/pm	Change	NM/pm	Change
			[%]		[%]
1.	Australia 2000	246		231	
	2015	383	56	380	65
2.	Austria 2000	129		91	
	2015	226	75	202	122
3.	Belgium 2000	238		274	
	2015	405	70	367	34
4.	Canada 2000	358		345	
	2015	393	10	399	16
5.	Denmark 2000	246		206	
	2015	405	65	419	103
6.	France 2000	331		280	
	2014	334	1	322	15
7.	Finland 2000	481		462	
	2015	999	108	938	103
8.	Germany 2000	169		117	
	2015	302	79	262	124
9.	Greece2000	151		77	
	2015	228	51	101	31
10.	Ireland 2000	217		194	
	2014	408	88	405	109
11.	Italy 2000	231		200	
	2015	288	25	270	35
12.	Japan 2000	71		49	
	2015	125	76	100	104
13.	Netherland 2000	260		272	
	2015	477	83	482	92
14.	New Zealand 2000	291		238	
	2013	344	18	342	44
15.	Norway 2000	262		204	
	2015	368	40	309	51
16.	Portugal 2000	162		121	
	2014	292	80	228	88
17.	Spain 2000	298		291	
	2015	394	32	401	38
18.	Sweden 2000	260		251	
	2015	398	53	436	74
19.	Switzerland 2000	312		274	
	2015	346	11	400	46
20.	UK 2000	217		192	
	2015	531	145	558	191
21.	USA 2000	330		325	
	2015	557	69	606	86

**Table 3:** Comparison the population of people aged 55–74 and the total populations of the eight largest analysed developed countries (actual numbers, both sexes, neurological mortality, population (in millions)). Odds ratios (OR) of total population to neurological mortality (NM). Based upon WHO [26] data.

	Population of 55–74 Years Old		Total Population		Total NM
Country	2000–2015	Change	2000–2015	Change	Odds Ratio
		[%]		[%]	
**Canada**					
Neurological mortality:	2649–3652	+38	19,293–35,091	+82	59%
Population (in millions):	0.496–0.761	+53	30.791–35.255	+14	
**France**					
Neurological mortality:	6236–5997	−4	40,594–71,543	+76	62%
Population (in millions):	10.628–13.956	+31	58.898–64.129	+9	
**Germany**					
Neurological mortality:	5790–9332	+61	22,543–73,310	+225	227%
Population (in millions:)	18.424–19.491	+6	82.188–81.687	−1	
**Italy**					
Neurological mortality:	5693–6542	+68	27,554–61,678	+124	110%
Population (in millions):	12.598–14.231	+13	56.924–60.731	+7	
**Japan**					
Neurological mortality:	4438–8099	+82	14,023–56,027	+299	300%
Population (in millions):	29.392–33.471	+14	125.612–125.319	−1	
**Spain**					
Neurological mortality:	3892–5007	+29	26,679–62,871	+135	104%
Population (in millions):	7.888–9.876	+25	40.174–46.410	+16	
**UK**					
Neurological mortality:	4650–9019	+94	24,601–103,550	+321	286%
Population (in millions):	11.065–13.792	+25	59.704–65.110	+9	
**USA**					
Neurological mortality:	21,818–48,047	+120	174,708–436,438	+150	120%
Population (in millions:)	42.666–67.380	+58	281.421–319.929	+14	

## Data Availability

Ethical approval was not required as the manuscript was based on published material in the public sphere.
